# Bone Marrow Adipose Tissue Deficiency Increases Disuse-Induced Bone Loss in Male Mice

**DOI:** 10.1038/srep46325

**Published:** 2017-04-12

**Authors:** Jessica A. Keune, Carmen P. Wong, Adam J. Branscum, Urszula T. Iwaniec, Russell T. Turner

**Affiliations:** 1 Skeletal Biology Laboratory, School of Biological and Population Health Sciences, Oregon State University, Corvallis, OR 97331,USA; 2 Biostatistics Program, School of Biological and Population Health Sciences, Oregon State University, Corvallis, OR 97331,USA; 3 Center for Healthy Aging Research, Oregon State University, Corvallis, OR 97331, USA

## Abstract

Bone marrow adipose tissue (MAT) is negatively associated with bone mass. Since osteoblasts and adipocytes are derived from the same precursor cells, adipocyte differentiation may occur at the expense of osteoblast differentiation. We used MAT-deficient *Kit*^*W/W−v*^ (MAT-) mice to determine if absence of MAT reduced bone loss in hindlimb-unloaded (HU) mice. Male MAT- and wild-type (WT) mice were randomly assigned to a baseline, control or HU group (n = 10 mice/group) within each genotype and HU groups unloaded for 2 weeks. Femurs were evaluated using micro-computed tomography, histomorphometry and targeted gene profiling. MAT- mice had a greater reduction in bone volume fraction after HU than did WT mice. HU MAT- mice had elevated cancellous bone formation and resorption compared to other treatment groups as well as a unique profile of differentially expressed genes. Adoptive transfer of WT bone marrow-derived hematopoietic stem cells reconstituted *c-kit* but not MAT in *Kit*^*W/W−v*^ mice. The MAT- WT → *Kit*^*W/W−v*^ mice lost cancellous bone following 2 weeks of HU. In summary, results from this study suggest that MAT deficiency was not protective, and was associated with exaggerated disuse-induced cancellous bone loss.

In long-duration spaceflight, the human skeleton adapts to microgravity by reducing the amount of bone in sites that are weight-bearing^1^. While new countermeasures have been implemented in a mission-specific manner, mitigating the metabolic changes of the skeleton in microgravity remains a challenge to the success of long-duration exploration class missions^2^,^3^.

An increase in bone marrow adipose tissue (MAT) is commonly observed with a decline in bone mass. This association has been observed in humans in iliac crest biopsies and with non-invasive use of magnetic resonance imaging, and in animal models for aging, postmenopausal osteoporosis^4^ and disuse^5^,^6^,^7^,^8^,^9^,^10^,^11^,^12^,^13^,^14^,^15^,^16^. Increased MAT was reported in humans following long-duration bedrest and in rodents during spaceflight^13^,^17^,^18^,^19^. Both osteoblasts and adipocytes differentiate from mesenchymal stem cells in the presence of specific transcription factors^20^,^21^,^22^,^23^. Since osteoblasts and adipocytes originate from the same progenitor pool within the bone marrow, it is possible that one cell type could be produced at the expense of the other^20^,^24^,^25^,^26^,^27^,^28^,^29^. However, a causal relationship has yet to be demonstrated. Thus, while conditions of disuse create a change in balance of cellular function within the bone marrow, it is not clear whether the concurrently observed infiltration of bone marrow fat actively contributes to bone loss.

We have previously identified *Kit*^*W/W−v*^ mice as deficient in bone marrow adipose tissue (MAT-) in weight-bearing bones^30^,^31^. These mice have a loss of function mutation in *c-kit,* a receptor tyrosine kinase, located on selective hematopoietic lineage cells^32^,^33^. Since an inverse relationship between MAT accumulation and bone mass has been observed, we hypothesized that if MAT plays a causative role, MAT- mice would be resistant to disuse-induced bone loss. To test this hypothesis, we utilized hindlimb unloading (HU) as a ground-based model for spaceflight^34^. The HU model produces skeletal changes in hindlimbs of rodents similar to those observed in astronauts following spaceflight^35^. We therefore compared the skeletal response of adult male WT mice and MAT- mice to HU.

## Results

### Experiment 1

**Figure 1:**
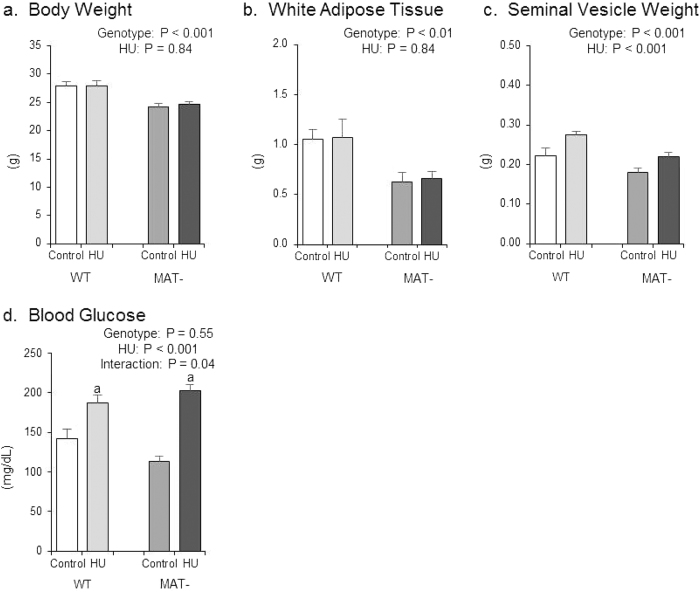
Effects of genotype, hindlimb unloading (HU) and their interaction on (**a**) body weight, (**b**) white adipose tissue weight, (**c**) seminal vesicle weight and (**d**) blood glucose. Two-way ANOVA: a, different from control mice within genotype. P-values significant at P ≤ 0.05. Mean ± SEM.

Experiment 1 evaluated the impact of MAT deficiency on the skeletal response to HU. The effects of genotype (WT and MAT-), treatment (control and HU), and their interaction on body weight, abdominal white adipose tissue (WAT) weight, seminal vesicle weight and blood glucose at necropsy are shown in Fig. 1. MAT- mice had a lower body weight than WT mice (Fig. 1a). No significant difference in body weight was observed in response to HU. MAT- mice had less abdominal WAT than WT mice (Fig. 1b). No significant difference in WAT weight was observed in response to HU. Seminal vesicle weight reflects testosterone levels in mice^36^. MAT- mice had lower seminal vesicle weight than WT mice (Fig. 1c) and HU resulted in greater seminal vesicle weight. Blood glucose was not different between genotypes (Fig. 1d). HU resulted in higher blood glucose concentrations, but MAT- HU mice experienced a greater increase in blood glucose.

**Figure 2:**
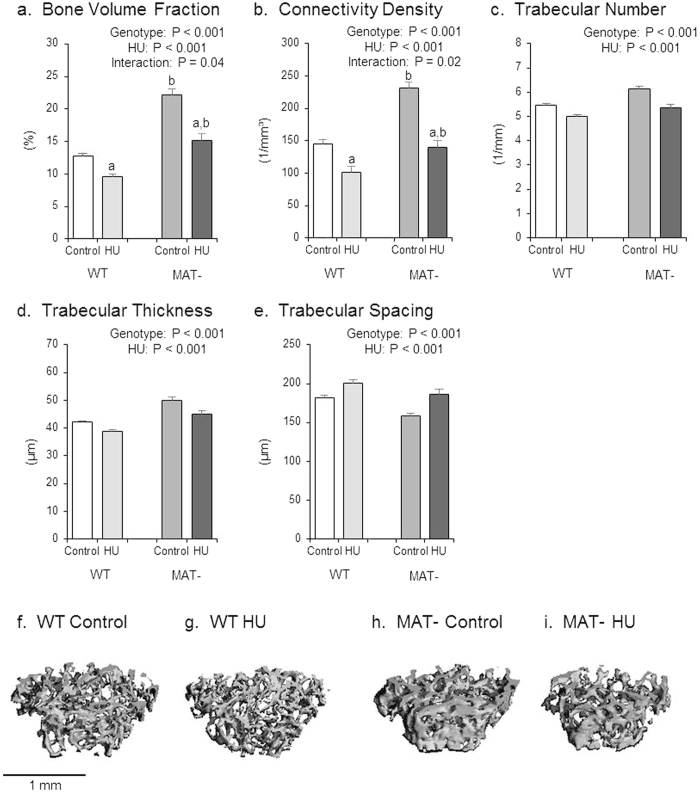
Effects of genotype, hindlimb unloading (HU) and their interaction on cancellous bone microarchitecture in the distal femur metaphysis. Shown are (**a**) cancellous bone volume fraction, (**b**) connectivity density, (**c**) trabecular number, (**d**) trabecular thickness, (**e**) trabecular spacing. Two-way ANOVA: a, different from control mice within genotype; b, different from WT mice within treatment. P-values significant at P ≤ 0.05. Mean ± SEM. Representative uCT images are show from (**f**) WT control, (**g**) WT HU, (**h**) MAT- control, and (**i**) MAT- HU mice. Images compiled by JAK.

The effects of genotype, HU and their interaction on cancellous bone microarchitecture in the distal femur metaphysis are shown in Fig. 2. MAT- mice had a greater bone volume fraction than WT mice (Fig. 2a). HU resulted in a lower bone volume fraction in both genotypes, but the MAT- mice experienced a greater reduction in bone volume fraction. MAT- mice had a greater connectivity density (Fig. 2b) than WT mice. HU reduced the connectivity density of both genotypes, but MAT- mice experienced a greater reduction. MAT- mice had greater trabecular number than WT mice (Fig. 2c). HU resulted in lower trabecular number. MAT- mice had greater trabecular thickness than WT mice (Fig. 2d). HU resulted in lower trabecular thickness. MAT- mice had lower trabecular spacing than WT mice (Fig. 2e). HU resulted in greater trabecular spacing. Representative images of microarchitecture in the femur metaphysis are shown in Fig. 2f–i.

The effects of genotype, HU and their interaction on cancellous bone microarchitecture in the distal femur epiphysis are shown in Supplementary Figure S1. Bone volume fraction was not different between genotypes (Fig. S1a). HU resulted in lower bone volume fraction. MAT- mice had lower connectivity density than WT mice (Fig. S1b). No significant difference in connectivity density was observed in response to HU. MAT- mice had lower trabecular number than WT mice (Fig. S1c). No significant difference was observed in trabecular number in response to HU. MAT- mice had greater trabecular thickness than WT mice (Fig. S1d). HU resulted in lower trabecular thickness. MAT- mice had greater trabecular spacing than WT mice (Fig. S1e). No significant difference was observed in trabecular spacing in response to HU.

To establish the direction of changes in bone microarchitecture following HU, the treatment groups were compared to controls sacrificed at the start of the experiment (baseline controls). The results are shown in Supplementary Table S1. In general, there was no difference in bone microarchitecture between control and baseline mice, the exceptions being cancellous bone volume fraction, connectivity density and trabecular number in the distal femur metaphysis of WT mice which were lower in the control mice. In contrast, most parameters differed between the HU mice and baseline mice. Specifically, cancellous bone volume fraction, connectivity density, trabecular number, and trabecular thickness were lower and trabecular spacing higher in the distal femur metaphysis of HU WT and HU MAT- mice. Also, cancellous bone volume fraction and trabecular thickness were lower in the distal femur epiphysis of HU WT and HU MAT- mice compared to baseline.

**Figure 3:**
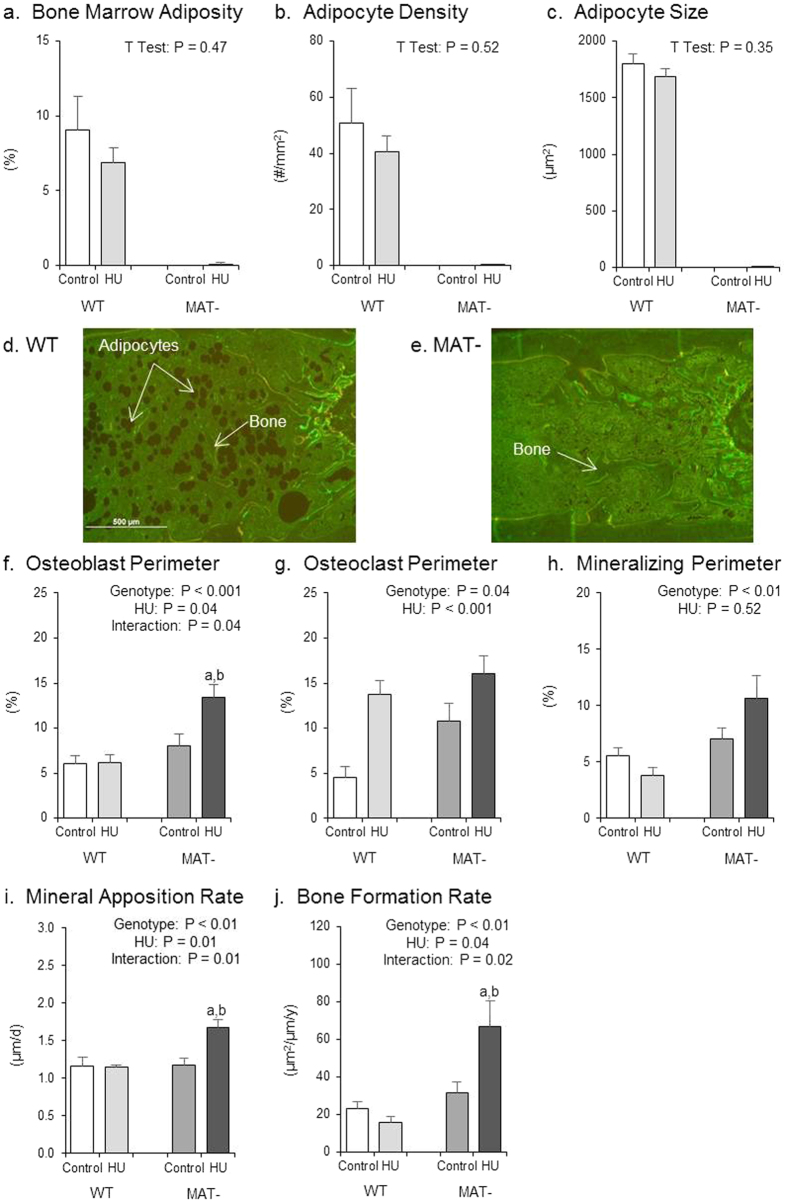
Effects of genotype, hindlimb unloading (HU) and their interaction on marrow adiposity and cancellous bone histomorphometry in the distal femur metaphysis. Shown are (**a**) bone marrow adiposity, (**b**) adipocyte density, (**c**) adipocyte size, (**d**) image showing presence of adipocytes in WT mouse, (**e**) image showing absence of adipocytes in MAT- mouse, (**f**) osteoblast perimeter, (**g**) osteoclast perimeter, (**h**) mineralizing perimeter, (**i**) mineral apposition rate, and (**j**) bone formation rate. Two-way ANOVA: a, different from control mice within genotype; b, different from WT mice within treatment. P-values significant at P ≤ 0.05. Mean ± SEM. Image scale is 500 μm at 4x. Images taken by JAK.

The effects of genotype, HU and their interaction on histomorphometry in the distal femur metaphysis are shown in Fig. 3. As expected, adipocytes in bone marrow were not detected in MAT- mice (Fig. 3a–c). The absence of adipocytes can be visibly appreciated in Fig. 3e. No significant differences were observed in bone marrow adiposity (Fig. 3a), adipocyte density (Fig. 3b) or adipocyte size (Fig. 3c) in response to HU in WT mice.

MAT- HU mice had greater osteoblast perimeter than WT HU mice (Fig. 3f). HU had no effect on osteoblast perimeter in WT mice, but resulted in greater osteoblast perimeter in the MAT- mice. MAT- mice had a greater osteoclast perimeter than WT mice (Fig. 3g). HU resulted in greater osteoclast perimeter. MAT- mice had greater mineralizing perimeter than WT mice (Fig. 3h). No significant difference in mineralizing perimeter was observed in response to HU. MAT- HU mice had greater mineral apposition rate than WT HU mice (Fig. 3i). HU had no effect on mineral apposition rate in WT mice, but resulted in greater mineral apposition rate in the MAT- mice. MAT- HU mice had greater bone formation rate than WT HU mice (Fig. 3j). HU had no effect on bone formation rate in the WT mice, but resulted in greater bone formation rate in MAT- mice.

**Figure 4:**
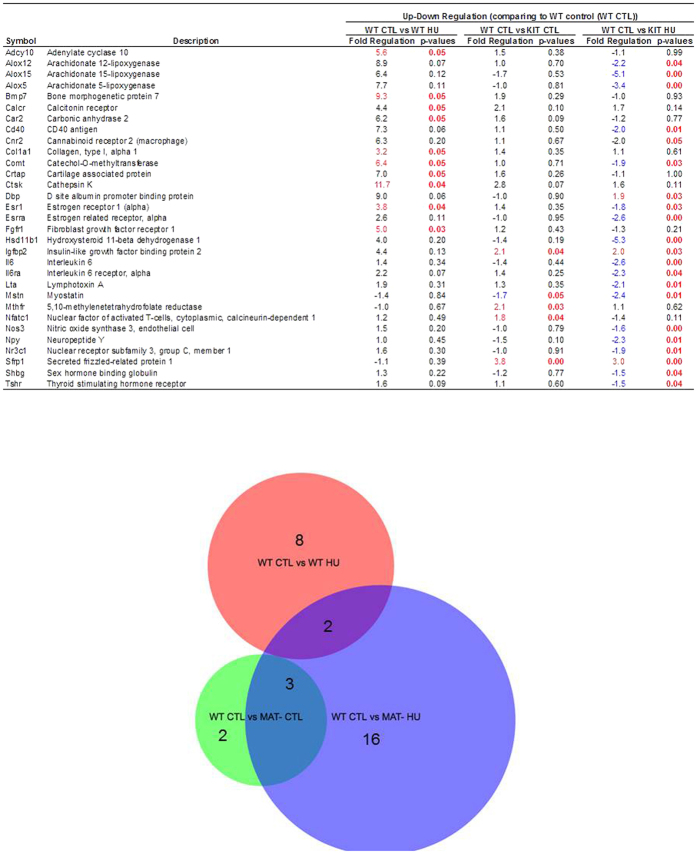
Relative expression of genes relating to osteoblast and osteoclast differentiation and function normalized to *Gapdh*. WT CTL mice were used to compare the effects of HU (red circle; 10 genes differentially expressed), MAT deficiency (green circle; 5 genes differentially expressed), and MAT deficiency after HU (blue circle; 21 genes differentially expressed).

Gene profiling was performed to evaluate whether HU leads to differential gene expression in WT and MAT- mice that may provide insight into the mechanisms mediating the observed differences in microarchitecture and cellular responses. The differential expression of genes related to osteoblast and osteoclast differentiation and function is shown in Fig. 4; the complete analysis of all 84 genes can be found in Supplementary Table S2. Compared to WT control mice, 5 genes (Igfbp2, Mstn, Mthfr, Nfatc1, Sfrp1) were differentially expressed in MAT- mice. Compared to WT control mice, 10 genes (Adcy10, Bmp7, Calcr, Car2, Col1a1, Comt, Crtap, Ctsk, Esr1, Fgfr1) were differentially expressed in WT HU mice. Compared to WT control mice, 21 genes (Alox12, Alox15, Alox5, Cd40, Cnr2, Comt, Dbp, Esr1, Esrra, Hsd11b1, Igfbp2, Il6, Il6ra, Lta, Mstn, Nos3, Npy, Nr3c1, Sfrp1, Shbg, Tshr) were differentially expressed in MAT- HU mice. Two of the genes that were differentially expressed in HU WT mice were differentially expressed in MAT- HU mice (Comt, and Esr1), but in opposite directions.

### Experiment 2

**Figure 5:**
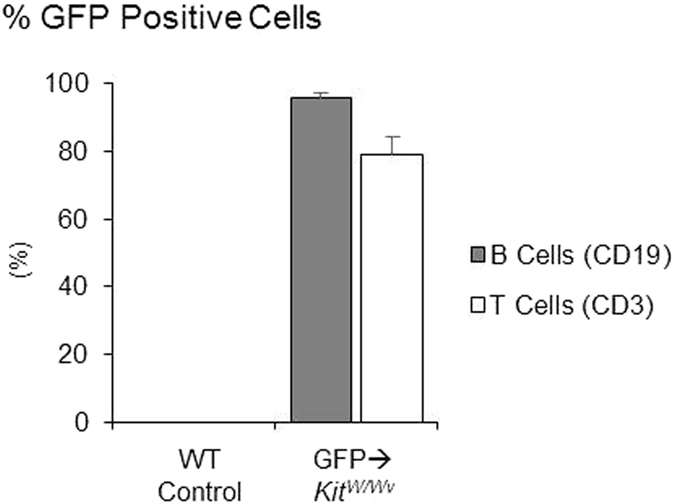
GFP-positive cells in WT control (no adoptive transfer) and *Kit*^*W/W−v*^ mice 8 weeks post adoptive transfer showing successful transfer of GFP-labeled HSC. Mean ± SEM.

**Figure 6:**
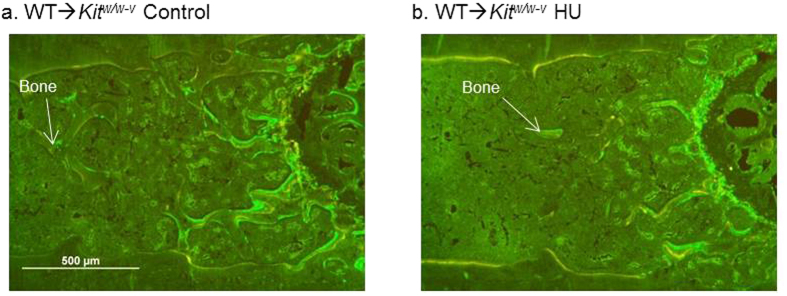
Images of histological sections from (**a**) WT→*Kit*^*w/w−v*^ control and (**b**) WT→*Kit*^*w/w−v*^ HU mice showing absence of adipocytes following adoptive transfer of WT HSC. Scale is 500 μm at 4x. Images taken by JAK.

A pilot study showed that transplanting WT hematopoietic stem cells (HSC) into *Kit*^*W/W−v*^ mice (WT→*Kit*^*W/W−v*^) reconstituted the hematopoietic compartment without restoring MAT. Therefore, Experiment 2 was performed to determine if HU-induced bone loss occurs with MAT deficiency in WT→*Kit*^*W/W−v*^ mice. The absence of MAT following adoptive transfer of WT HSC was confirmed in the present study (Fig. 5). To determine the extent of hematopoietic cell compartment reconstitution with donor cells in irradiated recipients, adoptive transfer of Green Fluorescent Protein (GFP)-positive HSC into irradiated recipients (GFP→*Kit*^*W/W−v*^) was concurrently performed. The percentage of donor-derived GFP positive B cells and T cells, two immune cell populations derived from HSC, were determined in the peripheral blood lymphocytes of recipient mice 8 weeks post adoptive transfer (Fig. 6). As expected, the control mice had no GFP-positive cells. In the GFP→*Kit*^*W/W−v*^mice, 95% of B cells and 79% of T cells were GFP positive.

**Figure 7:**
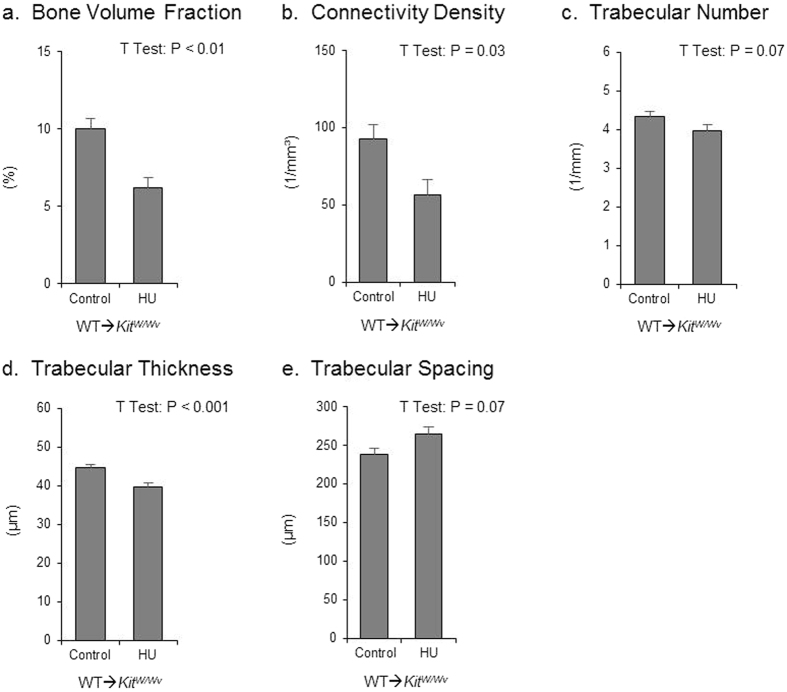
Effects of hindlimb unloading (HU) following adoptive transfer (WT→*Kit*^*w/w−v*^) on cancellous bone microarchitecture in the distal femur metaphysis. Shown are (**a**) cancellous bone volume fraction, (**b**) connectivity density, (**c**) trabecular number, (**d**) trabecular thickness and (**e**) trabecular spacing. T-test: P-values significant at P ≤ 0.05. Mean ± SEM.

The effect of HU on cancellous bone in the femur metaphysis of MAT- mice after adoptive transfer of WT HSC (WT→*Kit*^*W/W−v*^) is shown in Fig. 7. HU resulted in lower cancellous bone volume fraction (Fig. 7a), lower connectivity density (Fig. 7b), and lower trabecular thickness (Fig. 7d). No effect of HU was observed in trabecular number (Fig. 7c) or trabecular spacing (Fig. 7e).

Analysis of cancellous bone in the femur epiphysis of MAT- mice, who underwent adoptive transfer of WT HSC, is shown in Supplementary Figure S2. HU resulted in lower bone volume fraction (Fig. S2a). No effect of HU was observed in connectivity density (Fig. S2b), trabecular number (Fig. S2c), trabecular thickness (Fig. S2d) or trabecular spacing (Fig. S2e).

## Discussion

The present analysis is among the first to investigate the role of MAT in disuse-induced bone loss. The skeletal response to HU, an Earth-based model for spaceflight, was compared between normal mice and mice deficient in MAT (*Kit*^*W/W−v*^ mice). We hypothesized that an inability to produce MAT would provide protection from disuse-induced bone loss. This hypothesis stemmed from evidence that an increase in MAT during disuse was occurring at the expense of osteoblast formation. However, results from this study do not support our hypothesis. Indeed, MAT deficiency was associated with exaggerated bone loss.

The MAT- mice exhibited compartment-specific alterations in skeletal microarchitecture. In the distal femur metaphysis, MAT- mice had higher cancellous bone volume fraction and better bone quality (higher connectivity density, higher trabecular thickness, higher trabecular number and lower trabecular spacing) compared to WT mice. A different pattern was observed in the femur epiphysis where no difference between genotypes was detected in cancellous bone volume fraction, but the MAT- mice had lower trabecular number and higher trabecular thickness. Compared to cancellous bone in the metaphysis, which is highly sensitive to systemic factors that regulate mineral homeostasis (e.g., gonadal hormones), cancellous bone in the epiphysis experiences higher strain energy levels during weight bearing, suggesting a more important mechanical role, and exhibits lower bone loss in response to gonadectomy or aging^37^,^38^,^39^. The potential for site-specific differences in regulation illustrate the importance of evaluating multiple skeletal sites.

Histomorphometric analysis provides insight into the cellular basis for the microarchitecture in the metaphysis. Specifically, bone formation was higher in the MAT- mice compared to WT mice. These results support the concept that an inability to produce MAT may increase the production of osteoblasts^24^,^40^,^41^,^42^. Interestingly, osteoclast perimeter was also higher in MAT- mice. Presumably, the unbalanced bone turnover responsible for a higher cancellous bone volume fraction in MAT- mice is due, in part, to the previously described defect in osteoclast activity in these mice^43^.

Two weeks of HU resulted in reduced cancellous bone volume fraction in the metaphysis in both WT and MAT- mice, but the reduction was greater in MAT- mice. HU also resulted in changes in bone microarchitecture consistent with deterioration in bone quality (decreased connectivity density, trabecular number, trabecular thickness, and increased trabecular spacing). HU resulted in higher osteoclast perimeter in WT mice without altering osteoblast perimeter, a result consistent with the effect of spaceflight on biochemical markers of bone turnover is astronauts^44^,^45^,^46^,^47^. HU resulted in higher bone formation in the metaphysis of MAT-mice, as well as higher osteoclast perimeter. Thus, while HU resulted in higher osteoblast perimeter, it was insufficient to balance the higher rate of bone resorption, resulting in less cancellous bone due to unbalanced bone turnover.

Whereas 4 days of HU were associated with decreased expression of bone matrix proteins in BALBc and C3H mice^48^, we observed no decrease in expression of bone matrix proteins (Bglap, Col1a1, Col1a2) or alkaline phosphatase 14 days following unloading in WBB6F1/J mice. These findings are consistent with the normal osteoblast perimeter and bone formation in WBB6F1/J HU mice. The results further suggest that any suppression of bone formation following HU is transient, with the rebound occurring in response to increased bone resorption.

HU did not alter MAT levels, a finding that contrasts with the increases in MAT reported following spaceflight in rats^18^ and a previous HU study in mice^49^. The reason for this discrepancy is not clear. Few studies have evaluated effects of either spaceflight or disuse on MAT in mice. Thus, we cannot be certain that additional factors, including species, gender, age, duration of skeletal unloading and housing conditions, impact MAT accumulation during HU. The present study differs from earlier work in that mice in the present study were housed at thermoneutral temperature (32 °C). This may be relevant because MAT levels are reduced in mice by housing at room temperature^38^,^50^. Limitations of the present study include the relatively short duration of unloading and exclusive use of males. While the skeletons of male and female rats were reported to respond similarly to HU, gender comparisons have not been performed in mice^51^. Whatever the precise mechanism for the discrepancy in MAT accumulation during skeletal unloading, our results indicate an increase in MAT is not a prerequisite for HU-induced cancellous bone loss.

Spaceflight is associated with the development of subclinical diabetogenic changes in astronauts^52^. Whole body insulin resistance was observed within one day in growing HU rats^53^. In the present study, HU resulted in higher blood glucose levels, a response that was accentuated in MAT- mice. This suggests that MAT- mice have an impaired ability to regulate blood glucose levels which may be relevant to their excessive bone loss compared to WT mice as the skeletal response to mechanical loading is impaired in mice with hyperglycemia^54^.

Results from Experiment 1 indicate that an inability to produce MAT does not provide protection against disuse-induced bone loss. However, MAT- mice are also *c-kit* deficient. Kit signaling plays a role in regulating early osteoclast differentiation and mature osteoclast function^55^,^56^. Thus, impaired *c-kit* signaling may have impacted the skeletal response to HU. While adipocytes and osteoblasts are derived from mesenchymal stem cells, HSC express *c-kit*^57^. Adoptive transfer in Experiment 2 was successful in restoring WT hematopoietic lineage cells without restoring MAT. Two weeks of HU resulted in reduced cancellous bone in WT→*Kit*^*W/W−v*^ mice, indicating that MAT- mice with normal *c-kit* signaling are not protected against HU-induced cancellous bone loss.

Gene profiling adds further support to the conclusion that the underlying mechanisms mediating cancellous bone loss in the femur of HU MAT- and WT mice are not identical. There was little overlap in differentially expressed genes between HU WT and HU MAT- mice and where there was overlap (Comt, Esr1) the genes were differentially expressed in opposite directions. Particularly notable was the opposing differential response of estrogen receptor α (Esr1) in WT and MAT- mice to HU because estrogen receptor signaling plays an important role in the skeletal response to mechanical loading^37^. Also notable following HU was the genotype-specific (MAT- only) differential expression of genes related to fatty acid metabolism (Alox5, Alox10 and Alox12) and cytokine, neurotransmitter and hormone signaling (Comt, Hsd11b, Esr1, Igfbp2, Il6, Il6ra, Lta, Nos3, NPY, Nr3c1, Shbg, and Tshr). Further investigation is required to establish the significance of these differentially expressed genes in HU-induced bone loss.

In summary, analysis of bone microarchitecture, histomorphometry and gene expression revealed differences in the skeletal response to HU between MAT- and WT mice. Taken together, the results do not support the hypothesis that increased MAT contributes to disuse-induced bone loss in mice. MAT may actually attenuate disuse-induced osteopenia, perhaps by limiting the magnitude of increased bone turnover.

## Methods

The animals were maintained in accordance with the NIH Guide for the Care and Use of Laboratory Animals and the experimental protocol was approved by the Oregon State University Institutional Animal Care and Use Committee.

### Experiment 1

The purpose of this experiment was to evaluate the skeletal response to HU in WT and MAT-deficient *Kit*^*W/W−v*^ mice. Four-week-old male WBB6F1/J-*Kit*^*W*^*/Kit*^*W−v*^/J (*Kit*^*W/W−v*^or MAT-) mice and their WT WBB6F1/J littermates were purchased from Jackson Laboratory (Bar Harbor, ME, USA) and single housed in a 32 °C room for the duration of the experiment. Housing mice at 32 °C (thermoneutral temperature) has been shown to minimize resting energy expenditure^58^,^59^, which should compensate for the inability of mice to huddle (regulating body temperature) during HU. Furthermore, adaptation to room temperature housing was recently shown to result in cancellous bone loss in mice^38^.

At 16 weeks of age, the mice were randomized by body weight into one of six treatments (*n* = 10/group): WT baseline, WT control, WT HU, MAT- baseline, MAT- control, MAT- HU. Animals from the baseline groups were sacrificed. The next day, animals from the control and HU groups were transferred to unloading cages. The mice were unloaded for 2 weeks as described^34^. In brief, HU mice were placed in a restraint device, where the tail was cleaned with ethanol-soaked gauze and sprayed with a tincture of benzoin. A thin piece of traction tape was looped through a large paper clip, and then pressed along the sides of the mouse’s tail. Filament tape was wrapped around the tail in two locations to secure the traction tape: the base of the tail and 2.5 cm caudal. The paperclip end was looped through the clasp secured on the unloading apparatus. Mice were positioned in a 30° head-down tilt. All HU animals were provided with food and water *ad libitum*. The control groups were pair-fed to unloaded groups within genotype, and were given water *ad libitum*. Calcein injections (15 mg/kg; sc) were given 4 days and 1 day prior to termination to label mineralizing bone matrix. Mice were anesthetized then terminated using decapitation. The right femur from each mouse was placed in formalin for 24-hour fixation, then stored at 4 °C in 70% ethanol prior to sequential analysis by μCT and histomorphometry. The left femur was flash frozen in liquid nitrogen, then stored at −80 °C for RNA analysis. Body weight (g), abdominal WAT weight (g), seminal vesicle weight (g; an index of androgen levels) and blood glucose (mg/dL) were recorded at necropsy.

### Experiment 2

Mice were purchased and housed as in Experiment 1. WT littermates were used as donors of bone marrow. At 8 weeks of age, bone marrow transplant recipient mice were lethally irradiated (two split doses of 5 Gy each, 10 Gy total; Gammacell 220^60^ Co gamma irradiator) then, the following day, injected with purified HSC from donor WT mice. The purified HSC were prepared as follows: whole bone marrow cells were harvested from the femora and tibia of 12 donor mice. Lineage negative (lin^−^) cells were enriched from bone marrow cells using magnetic cell separation with MACS lineage cell depletion kit (Miltenyi Biotec Inc., Auburn, CA, USA). Enriched lin^-^ bone marrow cells were incubated with anti-CD117 (c-kit) and anti-Sca-1 antibodies (eBioscience, San Diego, CA, USA). HSC (Lin^−^Sca-1^+^c-Kit^+^) were purified from enriched lin^-^ by flow cytometry and single cell sorting^60^,^61^ using MoFlo XDP (Beckman Coulter, Indianapolis, IN, USA). Purified HSC were resuspended in saline, and 200 μl containing 1,000 donor HSC were injected into the tail vein of each irradiated recipient mouse. Tracking of cellular repopulation was performed with the use of HSC from GFP-expressing mice (GFP→ *Kit*^*W/W−v*^; *n* = 3) using the same protocol. The percentages of GFP-positive B and T cells in peripheral blood lymphocytes were measured by flow cytometry using B cell-specific (CD19) and T cell-specific (CD3) antibodies 8 weeks post adoptive transfer. Mice that did not undergo adoptive transfer were used as controls (*n* = 2).

At 16-weeks of age the mice were randomized by body weight into one of two treatments (*n* = 10/group): 1) WT→*Kit*^*W/W−v*^ control, 2) WT→*Kit*^*W/W−v*^ HU. Control and HU animals were then transferred to the unloading cages. Unloading and tissue collection were performed as described in Experiment 1.

### Micro-computed Tomography

μCT was used for nondestructive three-dimensional evaluation of cancellous bone volume and architecture. Femora were scanned using a Scanco μCT40 scanner (Scanco Medical AG, Basserdorf, Switzerland) at a voxel size of 12 μm × 12 μm × 12 μm (55 kV_p_ x-ray voltage, 145 μA intensity, and 200 ms integration time). Filtering parameters sigma and support were set to 0.8 and 1, respectively. The threshold value for evaluation was determined empirically and set at 245 (gray scale, 0–1000). Cancellous bone was evaluated in the distal femur metaphysis and epiphysis.

Assessment of cancellous bone in the distal femur metaphysis began 45 slices (540 μm in length) proximal to the growth plate, and included forty slices (480 μm in length) of cancellous bone. The entire cancellous bone compartment was evaluated in the distal femur epiphysis. Direct cancellous bone measurements included bone volume fraction (bone volume/tissue volume; volume of total tissue occupied by cancellous bone, %), connectivity density (number of redundant connections per unit volume, mm^−3^), trabecular thickness (mean thickness of individual trabeculae, μm), trabecular number (number of trabecular intercepts per unit length, mm^−1^) and trabecular spacing (distance between trabeculae, μm).

### Histomorphometry

The histological methods used have been previously described in detail^62^. In brief, distal femora were dehydrated in graded increases of ethanol and xylene, then embedded undecalcified in methyl methacrylate. Sections 4 μm thick were cut with a vertical bed microtome (Leica/Jung 2165) and fixed to slides with a dried precoated 1% gelatin solution. Mounted unstained slides were used for measurements of fluorochrome labels. For cell-based measurements, slides were stained with tartrate-resistant acid phosphatase and counterstained with toluidine blue (Sigma, St. Louis, MO, USA). All data were collected using the OsteoMeasure System (OsteoMetrics, Inc., Atlanta, GA, USA). The sampling site for the distal femoral metaphysis was located 0.25–1.25 mm proximal to the growth plate.

Static (cell-based) histological measurements include bone marrow adiposity (adipocyte area/tissue area; %), adipocyte density (#/mm^2^), adipocyte size (μm^2^), osteoblast perimeter (osteoblast perimeter/bone perimeter; %) and osteoclast perimeter (osteoclast perimeter/bone perimeter; %). Adipocytes were identified as large circular or oval-shaped cells bordered by a prominent cell membrane lacking cytoplasmic staining due to alcohol extraction of intracellular lipids during processing^63^. Osteoblast perimeter was determined as a percentage of total bone perimeter lined by plump cuboidal cells located immediately adjacent to the thin layer of osteoid in direct physical contact with bone. Osteoclast perimeter was determined as a percentage of cancellous bone perimeter covered by multinucleated cells with an acid phosphatase-positive cytoplasm (stained red).

Dynamic (fluorochrome) histological measurements include mineralizing perimeter (mineralizing perimeter/bone perimeter; %), mineral apposition rate (distance between two fluorochrome markers that comprise a double label divided by the 3 day label interval; μm/day), and bone formation rate (mineralizing perimeter multiplied by mineral apposition rate normalized to bone perimeter; μm^2^/μm/year).

### Gene Expression

Femora (n = 5/group) were pulverized with a mortar and pestle in liquid nitrogen, then further homogenized in TRIzol (Invitrogen, Carlsbad, CA, USA). Total RNA was isolated according to the manufacturer’s protocol, and mRNA was reverse transcribed into cDNA using SuperScript III First-Strand Synthesis SuperMix for qRT-PCR (Invitrogen). The expression of 84 genes related to osteoblast and osteoclast differentiation and function was determined using the Mouse “Osteoporosis” RT^2^ Profiler PCR Array (Qiagen, Valencia, CA, USA) according to the manufacturer’s protocol. Gene expression was normalized to *Gapdh* and relative quantification was determined by the ΔΔCt method using RT^2^ Profiler PCR Array Data Analysis software version 3.5 (Qiagen). Two of the twenty samples did not meet quality control standards for interpretation of array data and were excluded.

### Statistical Analysis

Means in Experiment 1 were compared between genotype and treatment groups using two-way analysis of variance (ANOVA). When significant interactions were present, t-tests were used to make two-group comparisons with “a” used to signify a difference from control mice within genotype and “b” used to signify a difference from WT mice within treatment. When non-significant interactions were present, group comparisons were made from two-way ANOVA with main effects for genotype and treatment. Means for bone marrow adiposity, adipocyte density and adipocyte size were compared using t-tests, since the absence of marrow adiposity in *Kit*^*W/W−v*^ mice made the use of two-way ANOVA invalid. Means among baseline, control and HU groups within genotype were compared using one-way ANOVA. When the ANOVA was significant, Dunnett’s test was used to make comparisons to the baseline group. Means in Experiment 2 were compared using t-tests

The required conditions for valid use of t-tests and ANOVA were assessed using Levene’s test for homogeneity of variance and the Anderson-Darling test of normality. When the assumption of equal variance was violated, Welch’s two-sample t-test was used for two-group comparisons^64^. When the normality assumption was violated, the Wilcoxon-Mann-Whitney test was used for two-group comparisons. Methods for maintaining false discovery rate at 5% were used to adjust for multiple comparisons^65^. Differences were considered significant at p ≤ 0.05. Data are presented as mean ± SEM. Data analysis was performed using RStudio version 0.98.1083.

## Additional Information

**How to cite this article:** Keune, J. A. *et al*. Bone Marrow Adipose Tissue Deficiency Increases Disuse-Induced Bone Loss in Male Mice. *Sci. Rep.* **7**, 46325; doi: 10.1038/srep46325 (2017).

**Publisher's note:** Springer Nature remains neutral with regard to jurisdictional claims in published maps and institutional affiliations.

## Supplementary Material

Supplementary Information
