# Supplementary material for: Bone Marrow Adipose Tissue Deficiency Increases Disuse-Induced Bone Loss in Male Mice
Source: Sci Rep. 2017 Apr 12;7:46325. doi: 10.1038/srep46325 (PMC5389344; doi:10.1038/srep46325)
Supplement: Supplementary Information [file srep46325-s1.pdf]

Bone Marrow Adipose Tissue Deficiency Increases Disuse-Induced Bone Loss in Male Mice

Jessica A. Keune[a], Carmen P. Wong[a], Adam J. Branscum[b], Urszula T. Iwaniec[a,c], and Russell T. Turner[a,c]*

[a]Skeletal Biology Laboratory, School of Biological and Population Health Sciences, Oregon State University, Corvallis, OR 97331,USA

[b]Biostatistics Program, School of Biological and Population Health Sciences, Oregon State University, Corvallis, OR 97331,USA

[c]Center for Healthy Aging Research, Oregon State University, Corvallis, OR 97331, USA

***Corresponding author**:
Russell T. Turner, Ph.D.
Skeletal Biology Laboratory
School of Biological and Population Health Sciences
Oregon State University
Corvallis, OR 97331
Tel: 541-737-9545
Fax: 541-737-6914
e-mail: russell.turner@oregonstate.edu

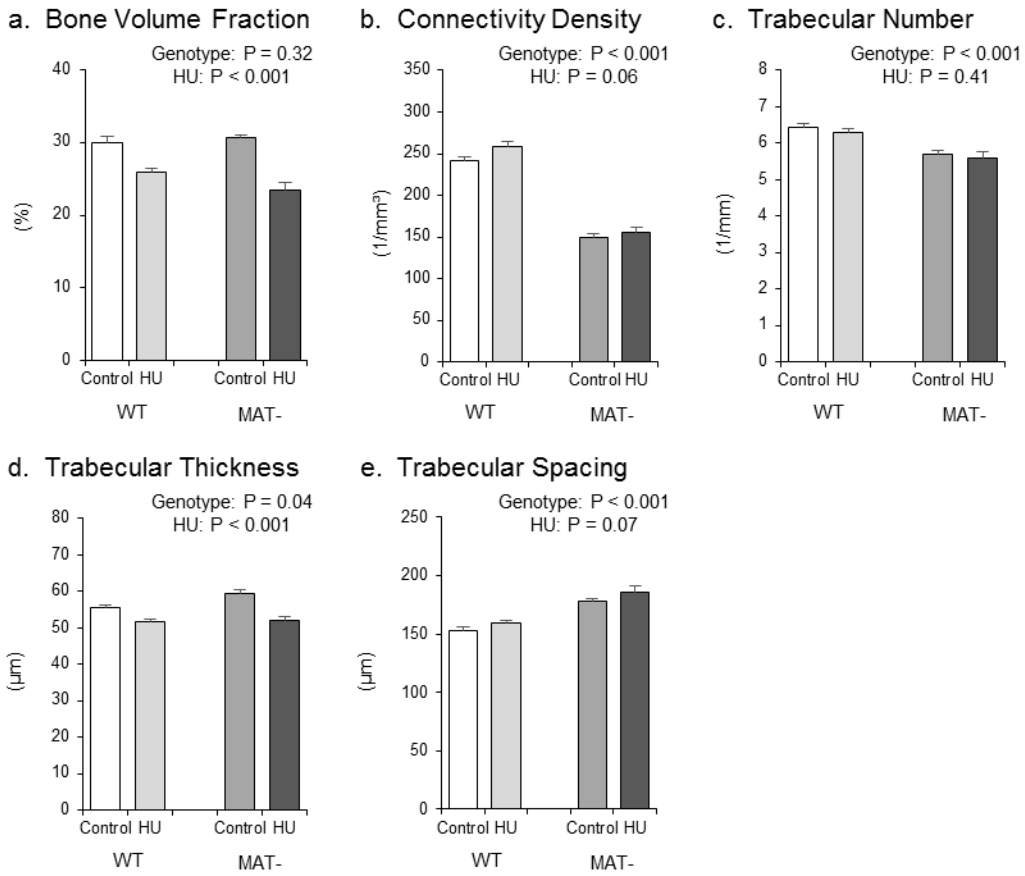

Figure S1.

Effects of genotype, hindlimb unloading (HU) and their interaction on cancellous bone microarchitecture in the distal femur epiphysis. Shown are a) cancellous bone volume fraction, b) connectivity density, c) trabecular number, d) trabecular thickness and e) trabecular spacing. Two-way ANOVA. P-values significant at P ≤ 0.05. Mean ± SEM.

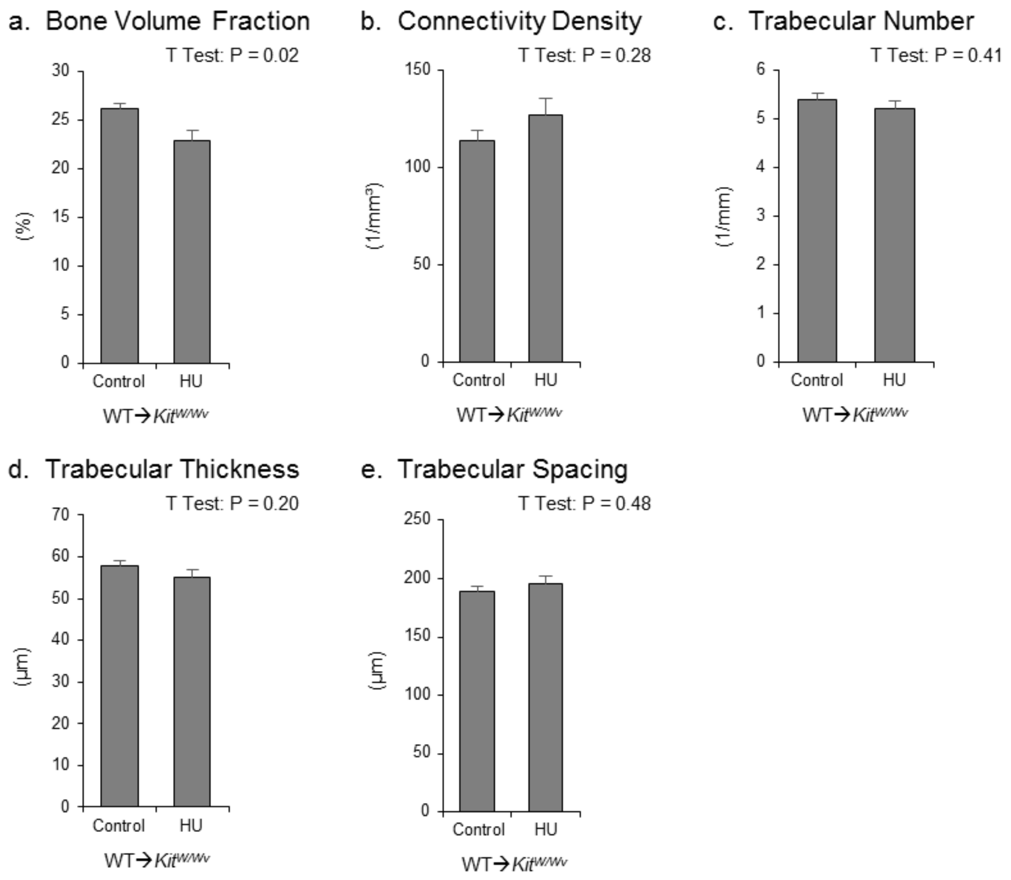

Figure S2.

Effects of hindlimb unloading (HU) following adoptive transfer on cancellous bone microarchitecture in the distal femur epiphysis. Shown are a) cancellous bone volume fraction, b) connectivity density, c) trabecular number, d) trabecular thickness and e) trabecular spacing. T-test: P-values significant at $P \leq 0.05$. Mean ± SEM.

Table S1. Effects of treatment on bone microarchitecture in the distal femur compared to animals sacrificed at baseline in Experiment 1. ANOVA and Dunnett's Test: P-values significant at P ≤ 0.05. Mean ± SEM.

	Wild-type Mice			ANOVA (P<)	MAT- Mice			ANOVA (P<)
	Baseline	Control	HU	Treatment	Baseline	Control	HU	Treatment
**Distal Femur Metaphysis** (cancellous bone)								
Bone Volume/Tissue Volume (%)	15.4 ± 0.5	12.8 ± 0.4*	9.5 ± 0.4*	**0.00**	21.5 ± 2.1	22.2 ± 0.9	15.2 ± 1.1*	**0.00**
Connectivity Density (1/mm³)	177.1 ± 11.9	144.4 ± 7.3*	101.8 ± 8.1*	**0.00**	221.2 ± 21.5	231.7 ± 9.1	139.3 ± 10.5*	**0.00**
Trabecular Number (1/mm)	5.7 ± 0.1	5.5 ± 0.1	5.0 ± 0.1*	**0.00**	6.0 ± 0.2	6.1 ± 0.1	5.4 ± 0.1*	**0.01**
Trabecular Thickness (µm)	45 ± 0	42 ± 0*	39 ± 1*	**0.00**	49 ± 2	50 ± 1	45 ± 1*	**0.03**
Trabecular Spacing (µm)	171 ± 3	182 ± 3	201 ± 4*	**0.00**	163 ± 10	159 ± 3	186 ± 6*	**0.03**
**Distal Femur Epiphysis** (cancellous bone)								
Bone Volume/Tissue Volume (%)	31.8 ± 0.4	30.0 ± 0.8	25.8 ± 0.6*	**0.00**	29.9 ± 1.1	30.6 ± 0.5	23.4 ± 1.1*	**0.00**
Connectivity Density (1/mm³)	240.5 ± 8.6	241.1 ± 4.7	257.8 ± 6.1	0.13	147.2 ± 7.4	149.4 ± 4.0	155.3 ± 5.8	0.61
Trabecular Number (1/mm)	6.5 ± 0.1	6.4 ± 0.1	6.3 ± 0.1	0.50	5.8 ± 0.2	5.7 ± 0.1	5.6 ± 0.1	0.75
Trabecular Thickness (µm)	57 ± 1	55 ± 1	52 ± 1*	**0.00**	58 ± 1	60 ± 1	52 ± 1*	**0.00**
Trabecular Spacing (µm)	151 ± 2	153 ± 3	160 ± 2	0.07	176 ± 5	178 ± 3	186 ± 5	0.33

* Different from Baseline
Control is pair fed to HU within genotype

Table S2. The expression of 84 genes related to osteoblast and osteoclast differentiation and function. Gene expression was normalized to *Gapdh*.

		Up-Down Regulation (comparing to wild-type control (WT CTL))					
		WT CTL vs WT HU		WT CTL vs MAT- CTL		WT CTL vs MAT- HU	
Symbol	Description	Fold Regulation	p-values	Fold Regulation	p-values	Fold Regulation	p-values
Adcy10	Adenylate cyclase 10	5.57	0.05	1.46	0.38	-1.07	0.99
Alox12	Arachidonate 12-lipoxygenase	8.93	0.07	1.03	0.70	-2.17	0.04
Alox15	Arachidonate 15-lipoxygenase	6.38	0.12	-1.68	0.53	-5.09	0.00
Alox5	Arachidonate 5-lipoxygenase	7.68	0.11	-1.04	0.81	-3.41	0.00
Alpl	Alkaline phosphatase, liver/bone/kidney	5.23	0.13	1.72	0.35	-1.26	0.67
Ar	Androgen receptor	7.92	0.06	1.77	0.26	-1.28	0.13
Bglap	Bone gamma carboxyglutamate protein	6.47	0.09	1.37	0.37	-1.49	0.26
Bmp2	Bone morphogenetic protein 2	5.75	0.12	1.61	0.31	-1.51	0.20
Bmp7	Bone morphogenetic protein 7	9.27	0.05	1.88	0.29	-1.04	0.93
Calca	Bone morphogenetic protein 7	4.87	0.06	1.17	0.49	-1.38	0.15
Calcr	Calcitonin receptor	4.43	0.05	2.14	0.10	1.74	0.14
Car2	Carbonic anhydrase 2	6.23	0.05	1.56	0.09	-1.25	0.77
Casr	Calcium-sensing receptor	3.91	0.17	1.30	0.44	1.04	0.89
Cd40	CD40 antigen	7.34	0.06	1.12	0.50	-1.96	0.01
Clcn7	Chloride channel 7	5.50	0.21	1.50	0.37	-1.26	0.66
Cnr2	Cannabinoid receptor 2 (macrophage)	6.27	0.20	1.05	0.67	-2.02	0.05
Col1a1	Collagen, type I, alpha 1	3.25	0.05	1.44	0.35	1.11	0.61
Col1a2	Collagen, type I, alpha 2	7.82	0.06	1.31	0.43	-1.20	0.52
Comt	Catechol-O-methyltransferase	6.38	0.05	1.01	0.71	-1.87	0.03
Crtap	Cartilage associated protein	7.00	0.05	1.61	0.26	-1.08	1.00
Ctsk	Cathepsin K	11.67	0.04	2.77	0.07	1.57	0.11
Cyp17a1	Cytochrome P450, family 17, subfamily a, polypeptide 1	3.93	0.08	1.18	0.55	-1.91	0.13
Cyp19a1	Cytochrome P450, family 19, subfamily a, polypeptide 1	4.34	0.07	1.14	0.55	-1.31	0.24
Dbp	D site albumin promoter binding protein	8.96	0.06	-1.02	0.90	1.95	0.03
Dkk1	Dickkopf homolog 1 (Xenopus laevis)	3.94	0.07	1.21	0.47	-1.78	0.07
Enpp1	Ectonucleotide pyrophosphatase/phosphodiesterase 1	4.63	0.06	1.45	0.07	-1.51	0.37
Esr1	Estrogen receptor 1 (alpha)	3.79	0.04	1.37	0.35	-1.81	0.03
Esr2	Estrogen receptor 2 (beta)	3.24	0.08	-1.12	0.81	-2.08	0.31
Esrra	Estrogen related receptor, alpha	2.65	0.11	-1.01	0.95	-2.63	0.00
Fgfr1	Fibroblast growth factor receptor 1	4.98	0.03	1.24	0.43	-1.26	0.21
Fgfr2	Fibroblast growth factor receptor 2	6.05	0.08	1.63	0.20	-1.26	0.30
Ghrh	Growth hormone releasing hormone	4.28	0.08	1.20	0.32	-1.23	0.31
Hsd11b1	Hydroxysteroid 11-beta dehydrogenase 1	4.04	0.20	-1.45	0.19	-5.32	0.00
Igf1	Insulin-like growth factor 1	6.54	0.08	1.74	0.11	-1.07	0.94
Igfbp2	Insulin-like growth factor binding protein 2	4.37	0.13	2.13	0.04	2.02	0.03
Il15	Interleukin 15	1.51	0.33	1.06	0.67	-1.54	0.14
Il6	Interleukin 6	1.42	0.34	-1.37	0.44	-2.57	0.00
Il6ra	Interleukin 6 receptor, alpha	2.19	0.07	1.38	0.25	-2.33	0.04
Itga1	Integrin alpha 1	1.90	0.20	1.43	0.31	-1.26	0.31
Itgb3	Integrin beta 3	2.02	0.11	1.78	0.25	-1.19	0.60
Lep	Leptin	2.07	0.19	1.08	0.55	-1.36	0.18
P3h1	Leprecan 1	3.76	0.19	1.07	0.75	-1.09	0.78
Lrp1	Low density lipoprotein receptor-related protein 1	2.40	0.14	1.23	0.43	-1.39	0.31
Lrp5	Low density lipoprotein receptor-related protein 5	2.30	0.07	1.01	0.92	-1.41	0.32
Lrp6	Low density lipoprotein receptor-related protein 6	2.20	0.06	1.18	0.47	-1.18	0.75
Lta	Lymphotoxin A	1.91	0.31	1.27	0.35	-2.05	0.01
Ltbp2	Latent transforming growth factor beta binding protein 2	2.28	0.06	1.20	0.48	1.01	0.98
Mab21l2	Mab-21-like 2 (C. elegans)	-1.74	0.73	1.51	0.12	1.01	0.88
Mmp2	Matrix metallopeptidase 2	-1.35	0.59	1.11	0.23	1.04	0.71
Mstn	Myostatin	-1.35	0.84	-1.72	0.05	-2.41	0.01
Mthfr	5,10-methylenetetrahydrofolate reductase	-1.01	0.67	2.11	0.03	1.12	0.62
Nfatc1	Nuclear factor of activated T-cells, cytoplasmic, calcineurin-dependent 1	1.15	0.49	1.78	0.04	-1.42	0.11
Nog	Noggin	1.50	0.38	1.02	0.73	-1.15	0.44
Nos3	Nitric oxide synthase 3, endothelial cell	1.54	0.20	-1.05	0.79	-1.58	0.00
Npy	Neuropeptide Y	1.02	0.45	-1.49	0.10	-2.27	0.01
Nr3c1	Nuclear receptor subfamily 3, group C, member 1	1.61	0.30	-1.03	0.91	-1.89	0.01
P2rx7	Purinergic receptor P2X, ligand-gated ion channel, 7	1.54	0.29	1.49	0.15	-1.24	0.23
Plod2	Procollagen lysine, 2-oxoglutarate 5-dioxygenase 2	1.35	0.32	1.13	0.52	1.07	0.60
Prl	Prolactin	1.31	0.56	-1.65	0.21	-1.56	0.28
Pth	Parathyroid hormone	-1.10	0.34	-1.05	0.70	-1.33	0.47
Pth1r	Parathyroid hormone 1 receptor	-1.59	0.39	1.53	0.13	1.38	0.33
Pthlh	Parathyroid hormone-like peptide	1.39	0.25	-1.17	0.29	-1.11	0.91
Runx2	Runt related transcription factor 2	-1.30	0.64	1.12	0.72	1.33	0.42
Sfrp1	Secreted frizzled-related protein 1	-1.09	0.39	3.78	0.00	2.98	0.00
Sfrp4	Secreted frizzled-related protein 4	1.17	0.29	1.51	0.16	2.03	0.10
Shbg	Sex hormone binding globulin	1.27	0.22	-1.15	0.77	-1.48	0.04
Sost	Sclerostin	-1.94	0.25	1.31	0.36	-1.49	0.11
Sparc	Secreted acidic cysteine rich glycoprotein	-1.36	0.43	-1.24	0.62	-1.17	0.07
Spp1	Secreted phosphoprotein 1	1.10	0.33	-1.26	0.37	-7.20	0.97
Stat1	Signal transducer and activator of transcription 1	1.17	0.30	-1.13	0.74	1.05	0.89
Tgfb1	Transforming growth factor, beta 1	1.00	0.41	1.14	0.63	1.03	0.93
Timp2	Tissue inhibitor of metalloproteinase 2	-2.02	0.82	1.34	0.21	1.16	0.40
Tnfaip3	Tumor necrosis factor, alpha-induced protein 3	-2.82	0.39	1.24	0.26	-1.06	0.77
Tnfrsf11a	Tumor necrosis factor receptor superfamily, member 11a	-1.06	0.87	1.79	0.13	1.40	0.17
Tnfrsf11b	Tumor necrosis factor receptor superfamily, member 11b (osteoprotegerin)	-1.05	0.46	1.09	0.45	1.08	0.56
Tnfrsf1b	Tumor necrosis factor receptor superfamily, member 1b	-1.84	0.56	1.17	0.59	-1.18	0.35
Tnfsf11	Tumor necrosis factor (ligand) superfamily, member 11	2.00	0.09	1.15	0.52	1.10	0.33
Tshr	Thyroid stimulating hormone receptor	1.60	0.09	1.10	0.60	-1.48	0.04
Twist1	Twist homolog 1 (Drosophila)	-1.28	0.70	-1.24	0.94	1.06	0.67
Vdr	Vitamin D receptor	-1.57	0.49	1.55	0.18	1.27	0.42
Vegfa	Vascular endothelial growth factor A	1.24	0.39	1.11	0.57	1.03	0.88
Wnt10b	Wingless related MMTV integration site 10b	-1.05	0.65	-1.23	0.65	-1.03	1.00
Wnt3a	Wingless-related MMTV integration site 3A	1.43	0.34	-1.35	0.43	1.25	0.33
